# Assessing the impact of authorisation process as a regulatory tool in the European REACH regulation: A study on improving occupational safety for applying companies

**DOI:** 10.1093/annweh/wxae032

**Published:** 2024-05-08

**Authors:** Holger-Lars Deubner, Gudrun Walendzik, Andreas Lüdeke, Urs Schlüter

**Affiliations:** Federal Institute for Occupational Safety and Health, Friedrich-Henkel-Weg 1-25, 44149 Dortmund, Germany; Federal Institute for Occupational Safety and Health, Friedrich-Henkel-Weg 1-25, 44149 Dortmund, Germany; Federal Institute for Occupational Safety and Health, Friedrich-Henkel-Weg 1-25, 44149 Dortmund, Germany; Federal Institute for Occupational Safety and Health, Friedrich-Henkel-Weg 1-25, 44149 Dortmund, Germany

**Keywords:** AfA, application for authorisation, authorisation, occupational safety and health, RAC, REACH, risk assessment, risk management, SEAC, SVHC, worker

## Abstract

This study looks into the effectiveness of the authorisation procedure as a regulatory instrument within the framework of the European REACH regulation. It highlights its impact on enhancing occupational safety and health for both applicants and companies utilising the substances. This procedure encompasses manufacturers, importers, and downstream users of substances, as well as representatives of foreign manufacturers who are also eligible to seek authorisation. When applying for authorisation, the ECHA Risk Assessment Committee (RAC) assesses the risks associated with the intended uses of the substance, including the appropriateness and effectiveness of the Occupational Conditions (OCs) and Risk Management Measures (RMMs) described in the application and the risks posed by potential alternatives. If the RAC determines that the OCs/RMMs are inadequate for managing or controlling the risk, or if the measures to protect workers are deemed insufficient, it may recommend additional measures to enhance occupational safety and health or environmental protection. The 398 processed Applications for Authorisation (AfA) that have been submitted to date were examined to determine these recommended measures, categorised as Conditions for use, Monitoring arrangements, and Recommendations for Review Reports. Overall, a significant improvement concerning occupational safety and health seems necessary, as indicated by the large number of measures recommended by the ECHA Committee for Risk Assessment (RAC) and ECHA Committee for Socio-economic Analysis (SEAC) or supplemented by the European Commission. In addition to the proposed measures, a short assessment provided by the committees as to whether the operational conditions and risk management measures are adequate in controlling the risks is also included in the study.

**What’s Important About This Paper?**

There is a gap in knowledge about how the European REACH Regulation has influenced occupational safety and health (OSH) through the authorisation process. To fill this gap, this study examined the applications for authorisation submitted to date, and found that many recommendations were made to improve OSH during the authorisation process. While more applications were found to have appropriate controls over time, there remains a need to improve OSH in European workplaces and the authorisation process has a role in this effort.

## Introduction

The REACH Regulation (Regulation (EC) No 1907, 2006) was introduced in 2006 among others to provide a framework for substitution of substances of very high concern (SVHCs) with safer alternatives, where feasible and socio-economically viable (ECHA, 2023). The REACH authorisation process is central to this framework, as it allows in a multistage process for the identification of SVHCs and their inclusion in Annex XIV of the REACH Regulation (List of Substances Subject to Authorisation). The main goal of the authorisation process is to replace SVHCs where technically possible with less dangerous alternatives, substances as well as production technologies, and to ensure that the use of these substances is properly controlled throughout the whole life cycle. The authorisation process also considers the risks, appropriateness, and effectiveness of the risk management measures. This study examines how the REACH authorisation process can contribute to occupational safety and health (OSH) management by applicants and companies using the substances, and thus complements the specific OSH regulation (Chemical Agents Directive (Directive 98/24/EC); Carcinogens, Mutagens, or Reprotoxic Substances Directive (Directive, 2004/37/EC)).

The so far limited research in this area between REACH and OSH often relates to topics such as exposure research, risk control, and the effectiveness of Operational Conditions (OCs) and Risk Management Measures (RMMs) (Zweetsloot et. al. 2011; Ott 2014; Hasle et. al. 2017; Cherrie et. al. 2020; Viegas et. al. 2020), while studies on occupational safety and health in the REACH context remain rare.

A previous study of the Federal Institute for Occupational Safety and Health (BAuA) on the risk reduction potential of REACH from 2018 contains first indications for requested improvements of the OCs/RMMs due to the Applications for Authorisation (AfAs) published so to date while underlining the potential of the authorisation process (Marx et. al. 2018). In contrast, a study of the downstream users in Sweden concludes that the REACH regulation has not had a major impact, but that the observed effects are positive (Schenk et. al. 2015). This is supported by similar studies that show that the REACH regulation as a regulatory instrument has not yet been able to fully develop its impact in the area of occupational safety and health throughout the first few years (Hammerschmidt et. al. 2014). However, results from more recent studies indicate a clear positive influence of the authorisation procedure and attest to its high functionality in terms of occupational safety (Dumke and Schlüter 2021; ECHA SOI 2021; Engel and Hanschmidt 2021; Reihlen et al. 2021; Reihlen, Jepsen and Wirth 2021; Schlüter, U. 2021; ECHA REF-9 2023).

## REACH authorisation system and application for authorisation (AfA)

In contrast to conventional chemical restrictions or bans, the authorisation requirement of the REACH Regulation enforces a prohibition contingent upon obtaining permission for the placement on the market and usage of substances listed in Annex XIV. This means that the use and marketing of substances listed in Annex XIV is generally prohibited unless an authorisation for a specific use, or several uses, have been granted based on an AfA. For each substance listed in Annex XIV a sunset date determines from which date on the placing on the market and the use of that substance shall be prohibited unless an exemption applies or an authorisation is granted, or an authorisation application has been submitted before the latest application date also specified in Annex XIV. Exemptions from the authorisation obligation are listed in Art. 56.

The use of substances listed on Annex XIV may be authorised in the EU if an applicant demonstrates in an AfA submitted to ECHA that the risks for the worker or the environment posed by the use of the substance are adequately controlled, or if the socio-economic benefits of its use outweigh the risk and no suitable alternatives exist.

During the Authorisation process, the applicant prepares an AfA for one or several uses and for one substance or a group of substances. The application is checked by ECHA for completeness and compliance of the data. After payment of the application fee, the AfA is ready for evaluation by the two committees, RAC (Committee for Risk Assessment) and SEAC (Committee for Socio-economic Analysis). As a part of this process, a public consultation is started by ECHA to collect information on potential alternative substances or technologies for the intended uses. In parallel, the opinion development of the committees RAC and SEAC starts resulting in a final opinion which—next to other information—takes also comments of the applicant into account. Finally, ECHA sends the opinion to the European Commission where a draft decision is prepared and adopted in the REACH Committee.

As per the latest information from ECHA (as of 15 March 2022), the three most common substances for which authorisations were sought are chromium trioxide (66 applications between 2015 and end of 2022), 4-(1,1,3,3-tetramethylbutyl)phenol, ethoxylated (53 applications between 2019 and end of 2022, only environmental concerns) and sodium dichromate (22 applications between 2015 and end of 2022). All chromium-containing compounds together (chromium trioxide, sodium chromate and dichromate, lead sulfochromate yellow, etc.) account for 115 applications, making up nearly half (46%) of all submissions and representing nearly 200 applicants and thousands of users (ECHA AfA 2022).

Of special importance for this study is, that as part of opinion development, RAC assesses the risks associated with the substance’s intended uses. These assessments are based on the hazardous properties of the substances specified in Annex XIV. This assessment encompasses the appropriateness and efficiency of the OCs/RMMs described in the AfA, as well as, if applicable, the risks posed by potential alternatives. Additionally, any third-party contributions related to the application will also be taken into consideration. If, in the opinion of RAC, the risk is not adequately controlled or the measures for the protection of workers or the environment are not appropriate and effective in limiting the risk, it may recommend additional measures to improve the occupational safety and health and/or protection of the environment. The proposed measures are included in the opinion of RAC and SEAC. The proposed measures can be adopted in the implementing decision of the Commission, which also has the right to add further measures or to change proposed measures.

All authorisation decisions have a time-limited review period. During this period, authorisation holders have to continue looking for a suitable alternative substance or technology that would make the use of the substance of very high concern (SVHC) unnecessary. If the authorisation holders do not succeed in this, they can submit a review report. This report must be submitted at least 18 months before the end of the review period. As part of the review reports, the authorisation holders should update all documents submitted in the original application that have changed and submit any other elements required by the conditions or monitoring arrangements of the authorisation decision.

## Methods

In this study, several sources were utilised to evaluate occupational safety and health in the context of the REACH authorisation process. These sources include the Chemical Safety Reports (CSR) and the list of OCs/RMMs from AfAs, the compiled RAC and SEAC opinions and the authorisation decision of the European Commission. All of these documents are available on the ECHA website, under the section ‘*Adopted Opinions and Previous Consultations on AfA*’ (ECHA, 2022). An example of an AfA for the use of chromium trioxide in chrome plating of valves used in light gasoline and diesel engines and heavy-duty diesel combustion engines (Mahle, 2018; Mahle RMM, 2018; Kadiķis, 2019; COM, 2020) serves as an illustration of these documents and the authorisation process.

In this study, the complete number of 398 decided authorisations (ECHA, 2022) were examined to assess the measures which are subject to granting authorisations (End-date of evaluation: November 2022). All authorisation decisions have an expiration date after which the authorisation is no longer valid. Until this date, authorisation holders have to continue looking for a suitable alternative substance or technology that would make the use of the substance of very high concern (SVHC) unnecessary. If the authorisation holders do not succeed in this, they can submit a review report. This report must be submitted at least 18 months before the end of the review period.

**Fig. 1.**
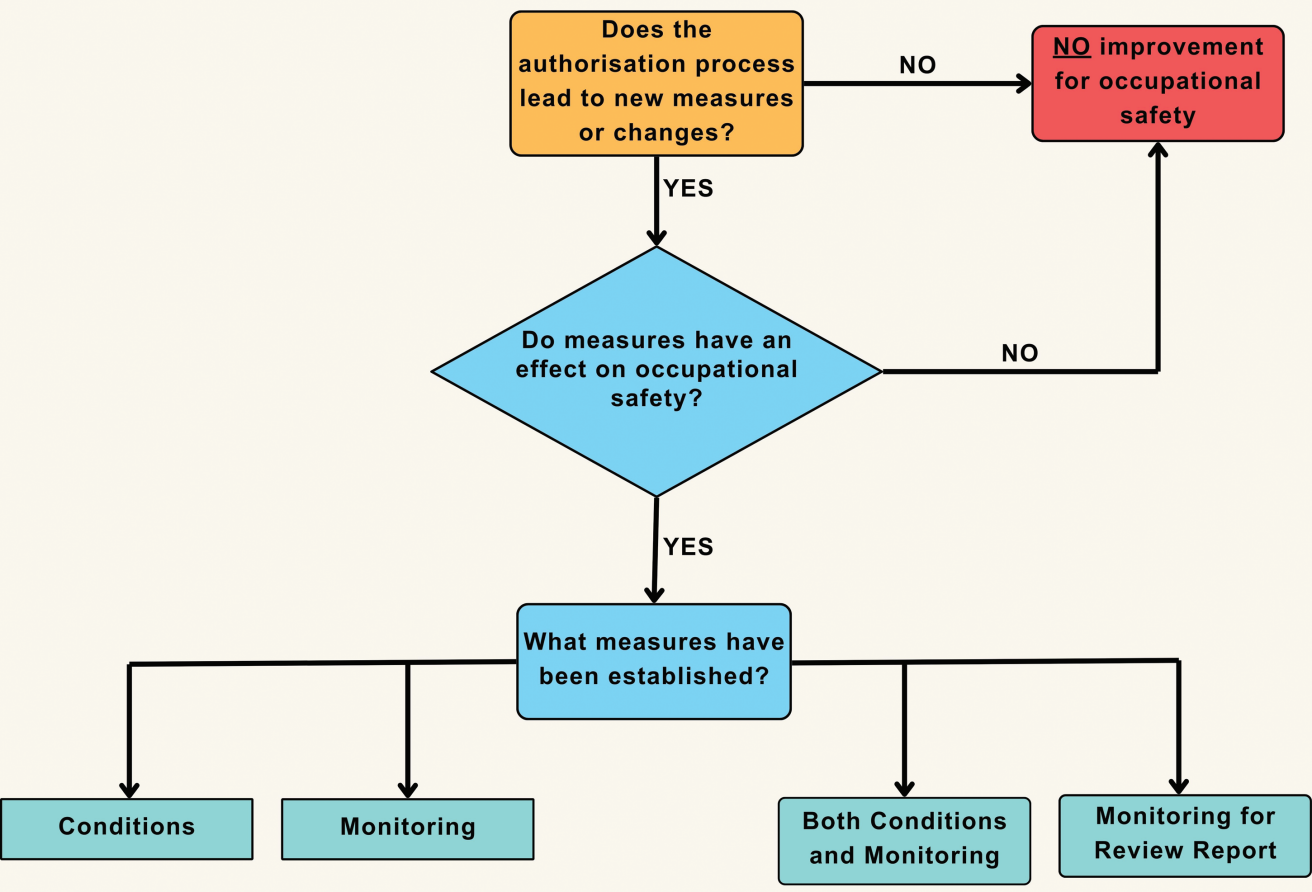
Decision tree for evaluating benefits for occupational safety and health in AfAs.

As part of the review reports, the authorisation holders should update all documents submitted in the original application that have changed and submit any other elements required by the conditions or monitoring arrangements of the authorisation decision. Conditions refer to technical changes and measures in processes, such as ventilation, the use of closed systems or wastewater treatment. Monitoring encompasses workplace monitoring (usually air monitoring) and employee biomonitoring. The procedure for decision making if the authorisation process leads to improvements in occupational safety and health is shown in Fig. 1.

In order to determine whether the authorisation process has improved occupational safety and health for companies, criteria must be established for evaluating such improvements. Due to the fact, that companies do not typically disclose any measures or improvements taken in preparing their applications, a direct link between implementation of risk management measures and the authorisation obligation cannot be established. However, very often the authorisation decision comes along with authorisation conditions which prescribe measures to improve occupational safety and health.

It needs to be underlined, that these conditions and monitoring arrangements need to be implemented by the applicants such that the health and safety benefits can be realised. In the following compliant behaviour of the applicants is assumed, as implementation of the measures is mandatory for the applicants. The national enforcement is not be examined in this study.

The first question arising in this decision tree (“Does the authorisation process lead to new measures or changes?”) can be answered by checking the Compiled Opinion of RAC and SEAC and the decision of the Commission with regard to whether measures were proposed, namely *additional conditions for the authorisation*, *monitoring arrangements for the authorisation* and/ or r*ecommendations for a possible review report*. The *Commission Implementing Decision* may specify the proposed measures in the context of the adopted decision.

The second question (“Does measures have an effect on occupational safety?”) relates to the impact of the measures on occupational safety and health. While for many of these measures a benefit for occupational safety and health is obvious (e.g. closed systems, local exhaust ventilation, protective barriers or spatial separation, reducing the number of workers exposed, reducing the time of exposure, wearing or improving PPE), some measures are more difficult to assess.

An example of these ambiguous measures are monitoring arrangements. While monitoring arrangements alone will not directly improve occupational health and safety, they can become relevant if phrases like ‘t*he information gathered via the measurements (referred to in points (a) and (b) and related contextual information) shall be used by the applicants to confirm the effectiveness of proposed RMM and OCs as well as to review annually the effectiveness of the RMM and OC in place and, if needed, to introduce measures to further reduce workplace exposure to chromium trioxide and emissions to the environment to as low a level as technically and practically feasible*’ (ECHA 2021) are added. This mandatory regular review of the OCs/RMMs based on the collected monitoring data in combination with the obligation to reduce workplace exposure to the feasible minimum, ensures a continuous process which, in addition to reducing emissions, has substantial impact on the protection of workers.

The classification of the measures as the third and final question in the decision tree (“What measures have been established?”) is also based on the information given in the respective chapters 7, 8, and 9 of the RAC and SEAC opinion and the decision of the Commission. These two documents already provide the four different categories and thus facilitate classification.

## Results and discussion

The RAC Committee has developed evaluation criteria for substances on Annex XIV such as for reference DNELs (*Derived No-Effect Level: derived exposure level below which the substance does not adversely affect human health*; for substances with a threshold of the toxicological endpoint) or reference dose–response relationships (for substances without a threshold of the toxicological endpoint) (ECHA EA 2023). This is because adequate control can only be demonstrated if a threshold for the relevant exposure and by this a DNEL (or PNEC for environmental concerns) can be determined for the relevant endpoint.

As a result, RAC cannot provide an opinion on whether acceptable exposure levels have been reached for non-threshold substances. However, even in the case of a non-threshold substance, RAC still assesses the appropriateness and effectiveness of proposed OCs/RMMs, and evaluate whether these are effective in limiting the risk based on the applicant’s exposure assessment, while also taking into account relevant community or national legislation and information from the public consultation.

## Risk management measures

**Fig. 2.**
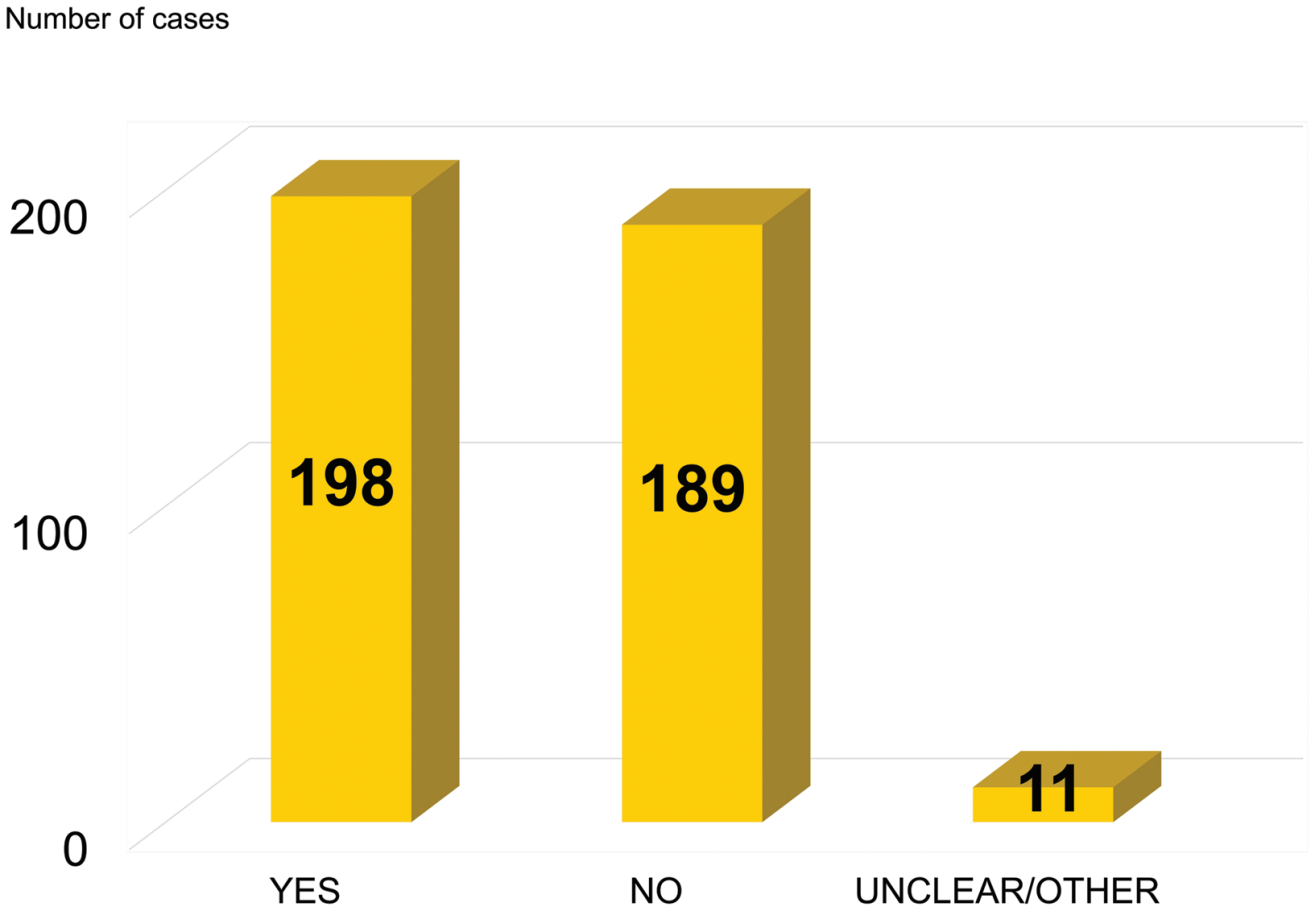
Case study of the 398 RAC assessment on effectiveness of the OCs/RMMs.

Following the above decision tree (Fig. 1) we have analysed whether RAC has identified deficiencies in risk control (“threshold substances”) or in OCs/ RMMs (“Non-threshold substances”) which may have led to recommendation of measures to address these deficiencies. Out of the 398 cases (ECHA 2022) analysed in this study, only 198 cases (50%) of the applications demonstrate OCs/RMMs that, according to RAC’s assessment, are effective in minimising the associated risks. This status only refers to the OCs/RMMs described by the applicant in the AfA. This implies that a significant number of 189 cases (47%) do not have appropriate and effective control measures in place to manage workplace risks at the time of the application (Fig. 2).

Concerning the effectiveness of operational conditions in ensuring sufficient control of risks over an extended period, a distinct trend can be observed if the 398 AfAs were divided into four waves of incoming AfAs (Table 1):

**Table 1.** Case comparison over time for effective control of risks (398 total cases). Comparison of cases with appropriate control of the risk (green) and not appropriate control of the risk (red). Based on compiled opinion of RAC and SEAC opinion, cases according to list of ‘Adopted opinions and previous consultations on applications for authorisation’ (ECHA 2022).

	Cases	Appropriate control of risk	No appropriate control of risk	Unclear cases
0001-01 to 0057-01	100	48	49	3
0057-02 to 0121-01	100	20	78	2
0121-02 to 0185-01	100	60	38	2
0185-02 to 0240-01	98	70	24	4

The figures indicate that at the initial stages of the authorisation process, only half of the applications demonstrate adequate control of the risks (48 out of 100 AfAs). The figures also indicate that a noteworthy improvement in more recent applications (100 and the latest 98 cases) has taken place, suggesting that the applying companies consecutively made progress in the field of occupational safety and health.

It should be noted that the assessment is made solely on the basis of the documents available, such as the CSR and the application for authorisation. The enforcement and actual status of the risk management prevailing in the plants cannot be assessed.

Over the years, a clear increase in the number of applications with adequate and effective risk control was observed by RAC. This either indicates an improvement in occupational safety and health within the companies or it shows that applicants adjusted their presentation of the workplaces in the applications for authorisation. Among others, differences due to unequal numbers of upstream and downstream applications are not taken into account, as well as learning effects on both sides in the practical handling of the process. An explanation for the notably low count of 20 cases with adequate risk control for the subset 0057-02 to 0121-01 cannot be deduced from the documents utilized.

## Effect of recommended risk management measures on occupational health and safety

**Fig. 3.**
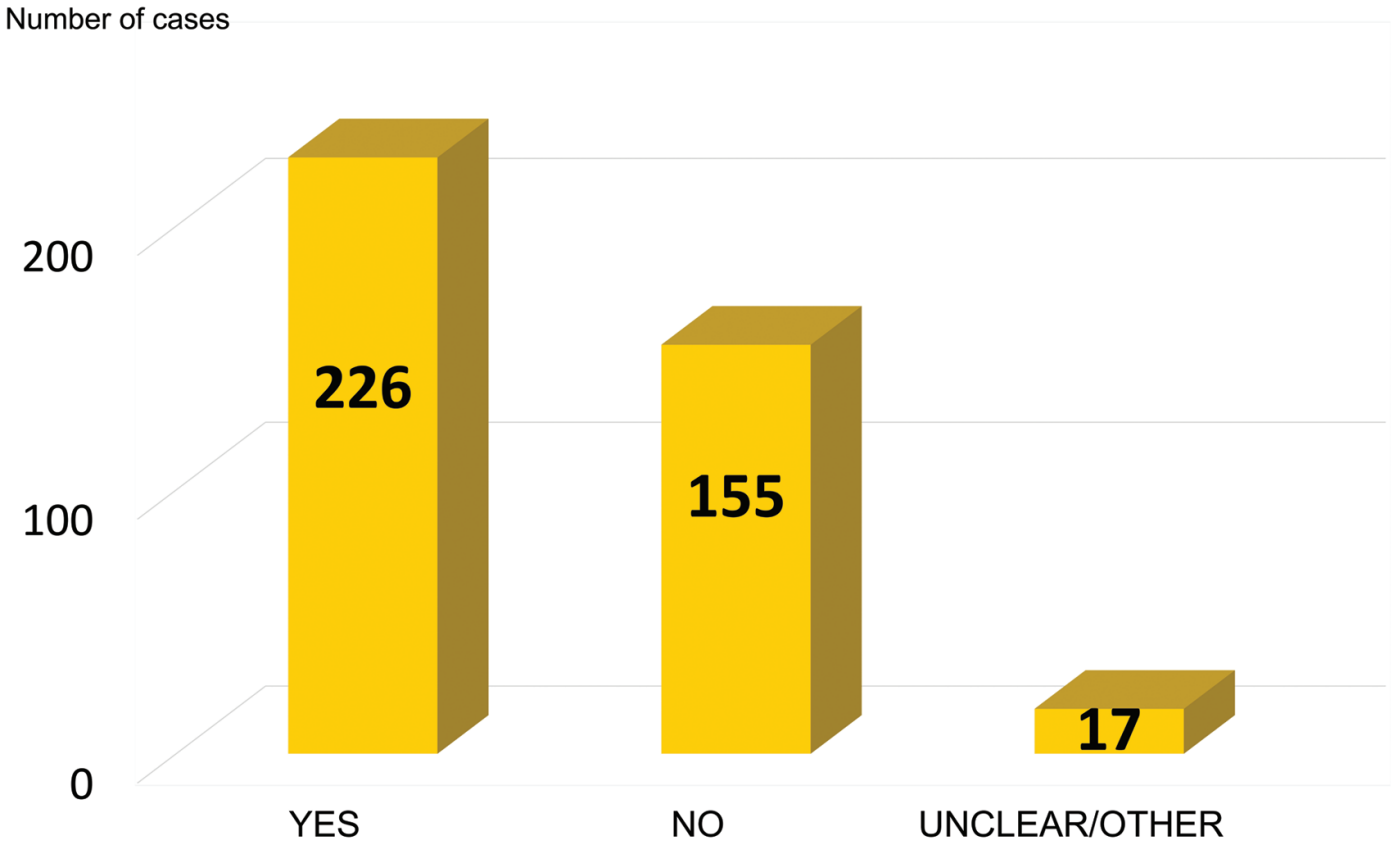
Comparison of the improvement of occupational safety and health through additional measures suggested by RAC and SEAC (Decision-making according to scheme shown in Fig. 1).

In section 7 “Proposed additional conditions for the authorisation, section 8 “Proposed monitoring arrangements for the authorisation” and section 9 “Recommendations for the review report” of the compiled RAC and SEAC opinion, additional measures for granting the authorisation are suggested. These measures are evaluated according to the decision tree shown in Fig. 1 to determine their influence on occupational safety and health as well as the workplace environment. Here, the applications can be split into three different scenarios: (a) a need for improvement in occupational safety and health, (b) no need for improvement in occupational safety and health, and (c) unclear or ambiguous cases such as withdrawn applications or cases without an available opinion from RAC and SEAC. The results of the evaluation are shown in Fig. 3.

Out of the total 398 applications for authorisation, RAC proposed additional measures in 226 cases (57%). This indicates that in approximately 6 out of 10 cases, the existing occupational safety and health measures of the companies were considered insufficient. This fact, in combination with the results presented in Figs. 2 and 3, show how often according to RAC, the control measures were not considered adequate. In only 155 of 398 cases (39%) no action was required and the workers were adequately protected. The number of 226 cases where additional measures for occupational safety and health were necessary, is in good agreement with the assessment on effectiveness of the OCs/RMMs in Fig. 2. However, the higher number (226 cases of additional measures versus 189 cases of not-adequate risk management) is due to the fact that although in some cases the measures are considered sufficient, RAC recommended or the Commission adopted additional measures to ensure the protection of workers over the entire authorisation period.

A good example thereof is AfA 0132-01 (ECHA 2019), an application for the use of chromium trioxide in electroplating. Here, RAC concluded that the OCs/RMMs described in the application are appropriate and effective in limiting the risk. Simultaneously, RAC proposes to implement an annual monitoring program for chromium (VI) to confirm the effectiveness of OCs/RMMs as well as to review annually the effectiveness of the OCs/RMMs in place. Furthermore, the authorisation holder shall ensure that the application of RMMs is in accordance with the hierarchy of control principles. The protection of workers is thus further increased or ensured for the review period, although the applying company has appropriate RMMs for limiting the risk. A distinct emphasis here is therefore on the further improvement of working conditions and not solely on the current appropriateness and effectiveness of the OC/RMMs as described in the CSR and the applicant’s control of the risk.

Although a need for improvement in occupational health and safety was identified in 226 cases (Fig. 3), RAC and SEAC recommended measures in significantly more cases (Table 2). In 285 cases (72%) RAC & SEAC suggested new measures, in 48 cases (12%) changes in already existing measures and in 58 cases (15%) no additional measures were needed or other options, like for example to refuse authorisation, were present. By adding the cases with new measures and cases with changes on existing measures, a total number of 333 cases (84%) emerges with intervention by RAC and SEAC. There is a discrepancy between 226 cases with a need for improvement in occupational health and safety and 333 cases with new or amended OCs/RMMs. This is due to measures proposed by the committees that have no impact on occupational health and safety. An example of this is requirements relating to environmental protection or monitoring of emissions.

**Table 2.** Overview of suggested measures by RAC and SEAC in their compiled opinion for the total 398 AfAs.

	Cases (total)	Percentage
New	285	71,6
Changes	48	12,1
No additional measures	58	14,6
No Authorisation	7	1,8

The 57% (226 cases; Fig. 3) in which occupational safety and health have potentially been improved (see Fig. 1) in combination with the 72% (285 cases, Table 2) of new measures are another important indicator for assessing the overall situation. Both figures demonstrate the need for general improvement on the company sites, with new measures addressing obvious gaps in the area of risk management and monitoring campaigns.

## Categories of measures proposed

This above finding is supported by the categories of measures proposed by RAC and SEAC in their opinions, and the implementing decision of the Commission. An overview of the types of measures is shown in Table 3.

**Table 3.** Type of measure suggested by RAC and SEAC in their compiled opinion. For 333 out of total of 398 cases in which RAC and SEAC proposed measures.

	Cases (total)	Percentage^*^
Conditions	30	9.0
Monitoring	78	23.4
Both Cond. and Monit.	154	46.2
Monitoring for review report	68	20.4

^*^1% missing for the three cases with production discontinued.

Here it is indicated that in almost half of the cases (154 cases, 46%) with recommended measures, both, conditions and monitoring are recommended. The exclusive application of monitoring arrangements is required in 78 cases (23%), only conditions in 30 cases (9%) and monitoring arrangements for the review report in 68 cases (20%).

As described above, not all monitoring arrangements for the review report represent an improvement for OSH. In the case mentioned, they became relevant because phrases similar to ‘*the authorisation holder shall use the information gathered via the measurements referred to (...) including the contextual information to regularly review the effectiveness of the OCs/RMMs and to introduce measures to reduce worker’s exposure to (...) as well as emissions to the environment to as low a level as technically and practically feasible*’ (ECHA 2021) were added. This supplement, with reference to annual review based on measurements at the working place has an immediate impact on working conditions and secures the applicant’s protection level over the entire review period.

Summarising the cases in Table 3 leads to 330 cases with suggested measures by RAC and SEAC. From these 330 cases, 232 (70%) include monitoring measures. This clearly underlines the great importance of monitoring and confirms the companies’ shortcomings in this area; usually the exposure assessment in applications for authorisation is based on modelling only. The RAC indicated from the beginning that for exposure assessment of Annex XIV substances (substances of very high concern) workplace monitoring (or environmental monitoring) is considered an adequate method.

**Fig. 4.**
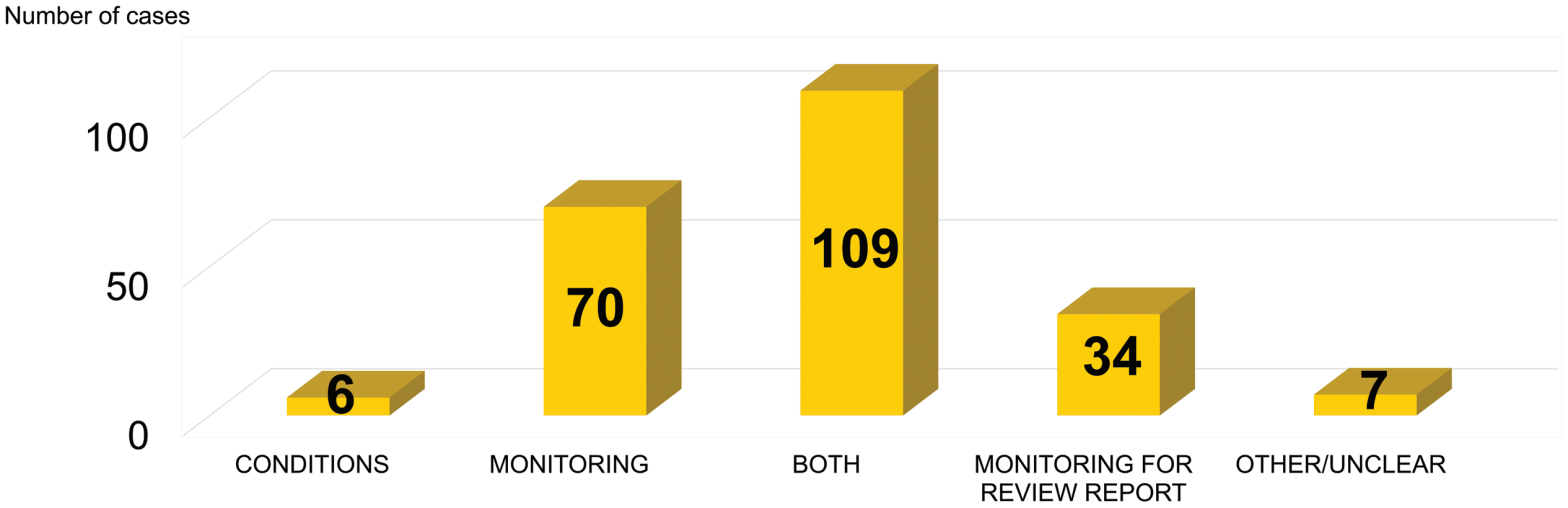
Type of measure suggested by RAC and SEAC in their compiled opinion for the cases with an identified improvement of occupational safety and health.

Looking just at the 226 cases with a need for improvement of occupational safety and health (Fig. 3), a very similar pattern emerges. The type of measure suggested by RAC and SEAC for cases with an improvement on occupational safety and health is shown in Fig. 4. Due to the high degree of individualisation of the conditions for each specific application and use, a detailed evaluation is not provided here.

Within this intersection, 213 cases (94%) include monitoring arrangements, which is a further increase compared to the findings above. At this point, a good agreement between the results in Table 3 and Fig. 3 is evident, which is expected since the database used for Fig. 4 is a subset of the data shown in Table 4.

**Table 4.** Additional or changes in measures of the European Commission for granting an authorisation based on the 253 cases where a decision of the Commission is present.

	Cases (total)	Percentage
Total	65	25.7
Conditions	45	17.8
Monitoring	12	4.7
Both cond. and monit.	8	3.2

Overall, the distribution is similar in cases where OSH has been potentially improved and furthermore in all cases, where measures have been recommended by RAC and SEAC. In both data sets, additional or changed conditions at the workplace are requested in more than half of the applications. Furthermore, additional monitoring arrangements are requested in nine out of ten cases.

The Risk Assessment Committee considers multiple measures, conditions, and monitoring, necessary in approximately every second case.

After the opinion development of RAC and SEAC (and the optional commenting by the applicant) the ECHA submits the compiled opinion to the European Commission where a draft decision is prepared and after discussion adopted in the REACH Committee. It is the responsibility of the European Commission to adopt, amend, or extend the recommended RMMs. In 65 (26%) of the total 253 cases in which a decision of the European Commission is adopted at the time of this examination, amendments or additional measures were included by the Commission. An overview of the OCs/RMMs added by the Commission is given in Table 4.

In about three of four cases considered, the Commission adopts the proposals of RAC and SEAC without changes concerning occupational safety and health and the OCs/RMMs. Unlike the measures proposed by RAC and SEAC presented in Table 3, many of the amendments address working conditions (45 cases, 69%). However, because the 253 examples where a decision of the Commission is present already had proposed measures, this number is not directly comparable to the measures arising from RAC and SEAC opinions. It just indicates that the Commission critically assesses the use of very hazardous substances through amendments and additions and further integrates measures itself to ensure the protection of workers handling SVHCs.

## Conclusions

It has been found that overall, a significant improvement in occupational safety and health in the context of the authorisation process seems necessary. One observation was that in a lot of cases deficiencies in the field of occupational safety and health were addressed in the ECHA opinions for AfAs and the European Commission decisions for granting authorisation. Consequently, RAC and SEAC have recommended a lot of measures of which most were taken up by the European Commission in decisions for authorisation. In addition, it can be seen from the data that the control of the risk for the use submitted by the applicant is not sufficient in a good half of the cases examined. Therefore, the ECHA Committees recommended further measures in more than eight out of ten cases, although the RMMs were often considered sufficient by RAC. The overall most frequently present measure for the protection of work are monitoring requirements, which are intended to confirm the effectiveness of the OCs/RMMs in place as well as to review them annually over the entire review period.

In summary, it seems justified to conclude that the use of SVHC needs to be improved all over Europe, and the AfA process has the potential to improve the safety of handling SVHCs in European workplaces.

## Data Availability

Data are taken from published “Adopted opinions and previous consultations on applications for authorisation”: https://echa.europa.eu/applications-for-authorisation-previous-consultations.
